# Individual differences in speech-on-speech masking are correlated with cognitive and visual task performance

**DOI:** 10.1121/10.0021301

**Published:** 2023-10-06

**Authors:** Andrew J. Byrne, Christopher Conroy, Gerald Kidd

**Affiliations:** 1 Department of Speech, Language and Hearing Sciences and Hearing Research Center, Boston University, Boston, Massachusetts 02215, USA; 2 Department of Biological and Vision Sciences, State University of New York College of Optometry, New York, New York 10036, USA

## Abstract

Individual differences in spatial tuning for masked target speech identification were determined using maskers that varied in type and proximity to the target source. The maskers were chosen to produce three strengths of informational masking (IM): high [same-gender, speech-on-speech (SOS) masking], intermediate (the same masker speech time-reversed), and low (speech-shaped, speech-envelope-modulated noise). Typical for this task, individual differences increased as IM increased, while overall performance decreased. To determine the extent to which auditory performance might generalize to another sensory modality, a comparison visual task was also implemented. Visual search time was measured for identifying a cued object among “clouds” of distractors that were varied symmetrically in proximity to the target. The visual maskers also were chosen to produce three strengths of an analog of IM based on feature similarities between the target and maskers. Significant correlations were found for overall auditory and visual task performance, and both of these measures were correlated with an index of general cognitive reasoning. Overall, the findings provide qualified support for the proposition that the ability of an individual to solve IM-dominated tasks depends on cognitive mechanisms that operate in common across sensory modalities.

## I. INTRODUCTION

The perceived spatial separation of multiple acoustic sources is an important binaural feature of real-world sound fields that can help listeners solve the “cocktail party problem” [refer to reviews in Middlebrooks *et al.* (2017)]. Many previous psychoacoustical studies using speech “targets” and simultaneous speech “maskers” [speech-on-speech masking (SOS)] have found that speech intelligibility performance improved as the degree of spatial separation of the maskers from the target increased over a certain range of values. The general performance advantage due to spatial separation of sources is referred to as “spatial release from masking” (SRM) and is well established in the human psychoacoustic literature. Studies in which the masker was Gaussian noise have reported much smaller benefits of spatial separation of sources (i.e., lower SRM) after taking into account monaural acoustic factors (e.g., “head shadow”), especially at target speech levels yielding typical high/conversational intelligibility (Kidd and Conroy, 2023).

Clayton *et al.* (2016) investigated several factors, including musicianship (cf. Swaminathan *et al.*, 2015), performance on a visual psychophysical task, and a battery of cognitive/executive function tests, that could potentially serve as predictors of individual differences in SRM for SOS masking. For a group of young-adult subjects, significant correlations were found between the amount of SRM and three predictor variables: musical training, performance on a visual attention task involving the tracking of multiple moving dots on a computer screen in the presence of other irrelevant/distracting moving dots (multiple object tracking; Pylyshyn and Storm, 1988), and a measure of cognitive/executive function. The executive function test that was significantly correlated with SOS performance was the digit span backwards (DSB) subtest of the Wechsler Adult Intelligence Scale (WAIS-5) battery. Of the cognitive subtests administered, which included other tests, such as nonverbal IQ (matrix reasoning), inhibition control, and cognitive flexibility, only the DSB test predicted SOS performance. An important part of the design in both the auditory and visual tasks employed by Clayton *et al.* was that the conditions were intentionally high in uncertainty and, therefore, presumably high in informational masking (IM) in the auditory domain and, by analogy, high in “IM” in the visual domain. The term “informational masking” commonly is applied to auditory task limitations (e.g., Kidd *et al.*, 2008b) but potentially could be generalized to visual tasks that are high in uncertainty (Zhang *et al.*, 2021).

Zhang *et al.* (2021) compared performance on auditory tasks high in IM [in contrast to energetic masking (EM); see Culling and Stone (2017) for a review] with both speech and non-speech stimuli to performance measured in a visual crowding paradigm considered to be a visual analog of auditory IM (i.e., determining how distinguishable a target object was in the peripheral field of vision from other adjacent distracting objects; Pelli, 2008). The Zhang *et al.* (2021) study found that performance across individuals was significantly *negatively* correlated for these auditory and visual tasks, indicating that, paradoxically, the subjects who were *better* in overcoming IM on the auditory task were *worse* on the high-IM visual task. This unexpected result led the authors to conclude—in contrast to the interpretation of the findings of Clayton *et al.* noted above—that an individual's “tolerance for clutter” extends only to one domain. This conclusion suggests that the effects of IM are in fact modality-specific, which may be considered paradoxical if it is thought that both tasks reflect the limitations of the same central mechanism (e.g., selective attention) that is influenced by uncertainty. It is also possible, however, that the auditory and visual IM tasks used by Zhang *et al.* (2021), while comparable in many ways, did not engage the same central mechanism(s). For example, speech intelligibility tasks tap linguistic processing to a degree that depends on factors, such as syntax and semantic content, that may be subject to influences not readily apparent in nonlinguistic visual tasks. Finding directly comparable tasks in the two modalities also is challenging due to differences in how the stimuli are perceptually segregated and how attention is engaged and applied in the specific tasks. The visual tasks used by Clayton *et al.* (2016) and by Zhang *et al.* (2021) were in fact quite different.

The finding by Clayton *et al.* (2016) that SOS masking performance, and the derivative SRM, were related to performance on a cognitive task thought to tax working memory—reversed digit recall—suggested that individual differences in “cocktail party problem” (CPP) listening performance may be governed by levels of processing higher in the auditory system than those usually attributed to “binaural analysis”—the process invoked to explain the “masking level difference” (MLD) for detection and the intelligibility level difference (SILD) for speech (Green and Yost, 1975). Some support for that interpretation may be found in other studies of cognitive factors in spatial hearing, especially those focused on the effects of age. For example, Fullgrabe *et al.* (2015) concluded that variations in the magnitude of SRM across age groups were associated more with cognitive differences than with binaural sensitivity changes due to aging. Older normal hearing adults also have been found to achieve lower performance than younger adults when the switching of spatial attention was required (Singh *et al.*, 2013), despite comparable performance for more basic SOS tasks. [It should be noted, however, that Wingfield *et al.* (2005) (review) warn that peripheral, central, and cognitive contributions to speech recognition may not be independent and that the general cognitive declines of aging, along with sensory losses and the cognitive effort required to compensate during competing speech, all interact to limit overall performance.] Similarly, children typically do not perform as well as adults for SOS intelligibility tasks (Hall *et al.*, 2002). Miller *et al.* (2018) suggested that the poorer performance in children may be due to limited development of cognitive skills and effort, while Newman *et al.* (2015) concluded that children as young as 4–6 years of age are susceptible to linguistic factors affecting IM in speech recognition. Thus, studies of subjects with declining or developing cognitive abilities suggest that poor performance is common on SOS tasks as is a reduced ability to exploit spatial separation of sources as a means to improve performance.

It has been suggested that the SRM pattern of performance typically found in SOS masking tasks is evidence that the listener establishes an attention-based “filter” tuned to spatial location (Marrone *et al.*, 2008). The idea is that such a filter would pass sounds emanating from the focus of attention in azimuth and attenuate sounds originating off axis in a manner somewhat analogous to the actions of an acoustic beamformer [see discussion in Kidd (2017)]. The sharper the spatial tuning, the better a listener can selectively attend to one talker and ignore competing talkers. Although group mean data clearly delineated these filter-like patterns of results, Marrone *et al.* (2008) observed individual differences not only with respect to the maximum amount of SRM that occurred (i.e., the dB difference between the colocated reference condition and the lowest spatially separated thresholds being referred to as the range of the filter), but also with respect to the sharpness of spatial tuning itself (i.e., the bandwidths and “attenuation” characteristics of individually fit filters). Furthermore, using SRM for SOS tasks is complicated by individual differences that vary for colocated and separated stimulus presentation due in part to the different segregation cues available in the two cases [refer to Kidd and Colburn (2017) and Byrne *et al.* (2022)]. It currently is not known whether these finer-grained analyses of SOS masking conditions could yield an improved understanding of individual differences in performance or the extent to which cognitive factors play a role in these differences.

Given that performance on selective attention tasks likely is modulated primarily by central, rather than peripheral, physiological structures (Zion Golumbic *et al.*, 2013; Puvvada and Simon, 2017) and that cognitive ability may be key, a natural question arises as to the extent to which these large intersubject differences characteristic of high IM are independent of the sensory modality that is stimulated. In our estimation, the limited evidence to date is inconclusive and in some cases contradictory. This lack of clarity may be due, in part, to the difficulty in finding tasks in the auditory and visual modalities that vary uncertainty in analogous and controlled ways and that may, therefore, produce different and comparable levels of IM.

The present study addressed the question of whether the large individual differences in masked speech identification performance for colocated and spatially separated sources were correlated with performance on a visual search task that was designed to be analogous to the SOS task in certain key respects (but not in all respects, as discussed below). Specifically, (1) uncertainty was varied in both auditory and visual tasks by manipulating the similarity between the target and maskers (cf. Kidd *et al.*, 2002). If it proved to be the case that individual performance was correlated across tasks, especially in high-IM conditions, it would be consistent with the proposition that susceptibility to IM reflects a domain general processing difference among subjects. In making this auditory-visual comparison, the key feature was the role of similarity in producing individual differences. (2) Spatial proximity/density of target and masker speech sources also was varied in a manner known to yield tuned responses in the auditory domain. A similar stimulus manipulation—physical proximity/density of target and masker objects—was performed in the visual domain (“proximity” in this case means the physical distance between visual elements; however, the key aspect of this stimulus manipulation is thought to be changes in the set size of candidate objects to be searched, as discussed further below).

In both stimulus manipulations—those intended to vary similarity and those varying the physical distance between the target and maskers—differences were expected in important aspects of task performance. Furthermore, the performance on a test of general cognitive ability—the Raven's Advanced Progressive Matrix test—and musical training were examined as predictors of auditory and visual task performance. These variables were central to the hypothesis that certain cognitive abilities play a significant role in listening performance (and potentially visual search performance) in highly complex and uncertain environments.

## II. GENERAL METHODS

### A. Participants

Eighteen individuals participated in both experiments of the current study, two of whom were the first and second authors, while the other individuals were paid volunteers. There were six males, 11 females, and one participant who did not specify gender, with an age range of 18–40 years and a median age of 22 years [mean (M) = 24.6, standard deviation (SD) = 6.5]. All participants had normal hearing based on pure-tone thresholds of 20 dB hearing level (HL) or better at octave frequencies from 250 to 8000 Hz. All participants self-reported that they had normal, or corrected-to-normal, vision and spoke English as their first and primary language. The experimental research procedures were approved by the institutional review board of Boston University, and all participants gave written informed consent.

### B. Cognitive measure and musicianship

All participants completed a task to measure their general cognitive reasoning ability, the Raven's Advanced Progressive Matrices (RAPM), which is considered a valid, non-verbal measure of analytic intelligence and abstract reasoning (Raven, 1936; Raven *et al.*, 1998) and which requires no task-specific prior knowledge to perform. This test requires the completion of intricate geometric designs with a missing piece, given eight options to choose from for each pattern, with the difficulty of the task generally increasing for each subsequent pattern presented (Carpenter *et al.*, 1990). After an explanation of the test using the first two items from the RAPM practice set (Set I), the participants were given 30 min to complete as many of the 36 patterns from Set II as possible, with their cognitive performance for the present study defined as the total correct answers given. RAPM scores ranged from 14 to 34, with a mean of 24.2 (SD = 5.9) and a median of 24.

The participants also completed a questionnaire describing their musical training background, with the definition of musical training including not only musical instruments, but vocalist training (e.g., choir) as well. The aspect of training that was chosen to define “musicianship” for the present study was the answer to the question: “How many total years of musical training do you have? (This includes group and private lessons or practicing on your own.).” In this context, the musical training of the participants ranged from 0 to 32 years, with a mean of 11.3 years (SD = 7.4) and a median of 11 years.

## III. EXPERIMENT 1: SPATIAL TUNING FOR MASKED SPEECH IDENTIFICATION

### A. Methods

#### 1. Stimuli

A closed-set matrix-style masked speech identification task was used (Kidd *et al.*, 2008a; Byrne *et al.*, 2022). The target utterances were five-word sentences using the fixed syntactic structure of name, verb, number, adjective, and object, e.g., “Sue saw two big shoes.” The speech corpus [refer to Kidd *et al.* (2008a)] included the natural production of eight exemplar words in each syntactic category, which had been recorded from 12 female talkers.

For each experimental trial, a target talker was randomly selected from the 12 options in the corpus, and the target sentence always began with the name “Sue.” For speech masker conditions, this name provided the target designation cue for the listener, who then must follow the voice of the target talker throughout the sentence. The remaining four words of the target sentence were randomly chosen from the eight options in each syntactic category, maintaining the syntactic structure described above. Two independent maskers were also constructed in a similar manner for each trial. The two masker talkers were randomly selected from the 11 remaining talker options (after excluding the target talker), and two sentences were constructed using random selection from the remaining words in each category conforming to the same syntactic order as the target sentence. All of the words in the three sentences were chosen randomly on each trial with the proviso that they were mutually exclusive. Note that these sentences preserve normal syntactic structure but are low in semantic value due to the random selection of words from a small set of exemplars on every trial.

There were three different types of maskers. For the “speech” masker condition, the masker sentences were presented naturally (i.e., as recorded and intelligible in quiet). A second “reversed” speech masker condition was created by time-reversing each individual word prior to sentence construction (i.e., the complete sentence was not time-reversed, and the syntactic order of the sentence was unaltered). The third masker condition used speech-spectrum-shaped speech-envelope-modulated noise (the “noise” condition), in which the broadband amplitude envelope was extracted from the words (using a Hilbert-transform) of each masker sentence and imposed on a speech-shaped noise, which was also derived from the masker sentence. Thus, the noise stimuli retained the same overall averaged spectrum as the original sentence and roughly the same broadband envelope. The target and masker stimuli were time-aligned such that only the beginning of each full sentence stimulus began simultaneously.

Stimuli level was defined by the root mean square of the waveform of each stimulus; the individual words were normalized in level prior to sentence construction. The composite masker level (both maskers added together) was held constant at 60 dB sound pressure level (SPL) across all presentations, while the level of the target was adapted to obtain target-to-masker ratios (TMRs). Despite the composite masker level being fixed, the two maskers on each trial were varied such that there was a level difference of 0–10 dB between the two maskers on each trial, with the level difference randomly chosen from a rectangular distribution. Thus, one of the two maskers typically ranged from 57 to 60 dB SPL, while the other masker was 50–58 dB SPL.

The use of this masker level difference rove was motivated by previous research revealing non-monotonic psychometric functions for colocated SOS tasks (Dirks and Bower, 1969; Brungart, 2001; MacPherson and Akeroyd, 2014; Byrne *et al.*, 2022). In all cases, performance declines as positive TMRs decrease and approach 0 dB but, in at least some listeners, can improve as the target level decreases below 0 dB and becomes noticeably quieter than the maskers (i.e., segregation by level allows the softer level signal—the target—to be intelligible). The level difference range used here was chosen based on pilot testing and served to reduce the feasibility of attending to the lower-level stimulus (cf. Byrne *et al.*, 2022).

All stimuli were generated at a sampling rate of 44 100 Hz, and RME (Haimhausen, Germany) Digiface USB sound cards were used for audio output. Sennheiser (Wedemark, Germany) HD280 Pro headphones presented the binaural stimuli, which were spatialized using non-individualized head-related transfer functions (HRTFs; Gardner and Martin, 1994). The azimuth of the target stimulus was always at 0° (directly forward), while the azimuths of the two masker stimuli were a manipulated variable, with symmetric angles of 0°, ±5°, ±10°, ±20°, and ±40° used in separate conditions. Compared to Marrone *et al.* (2008), these smaller spatial separations were chosen based on Srinivasan *et al.* (2016), which found that only a few degrees of separation in azimuth yielded large SRM for many normal hearing listeners. The elevation was fixed at 0° for all stimuli.

#### 2. Procedure

The experiment was implemented on PC-style computers using matlab (MathWorks, Natick, MA), and participants were tested individually in Industrial Acoustics Company (North Aurora, IL) double-walled sound-attenuating chambers to reduce ambient noise and distractions. The task of the participant was to identify the words spoken by the target talker on each trial. Each participant was explicitly told that the target would always appear to emanate from directly in front of them, while the maskers could either also be forward or be “off to the sides” to various degrees. After each presentation, a graphical user interface (GUI) displayed the matrix of possible words, and the participant was required to make a selection in each syntactic category in the same order as the sentence constructions. (The cue word “Sue” was always pre-selected.) Correct answer feedback was only shown after all responses for that trial were registered.

A one-down, one-up adaptive tracking procedure was used to vary the level of the target to estimate the 50% correct point on the psychometric function. The tracking step size was fixed at 3 dB, with the participant needing to correctly identify all words in the target sentence (save the preselected cue word) to decrease TMR (increase difficulty). Each block of trials consisted of at least 24 trials and at least eight tracking reversals, and the block terminated after both of those conditions were met. The first two or three reversals were discarded, resulting in an even number of reversals for averaging, which was used to define the threshold TMR for each block. The first block for all conditions started with a TMR of 12 dB, while the second block for each condition started approximately 12 dB above the threshold of the first block (with a maximum starting TMR of 12 dB).

Experiment 1 consisted of 30 blocks, with two blocks for each of the three masker types and five masker separations. A complete set of each condition (15 blocks) was obtained prior to any repetition of conditions, and the order of the conditions within each set was randomized. Participants ran the experiment self-paced, typically over the course of two or three 2-h sessions, along with the task of experiment 2 (described later), with the order of each task (experiment 1 or 2) for each session counterbalanced across sessions. The total time required to complete experiment 1 was approximately 3 h, not including participant break periods.

### B. Results and discussion

#### 1. Spatial tuning functions

**FIG. 1.**
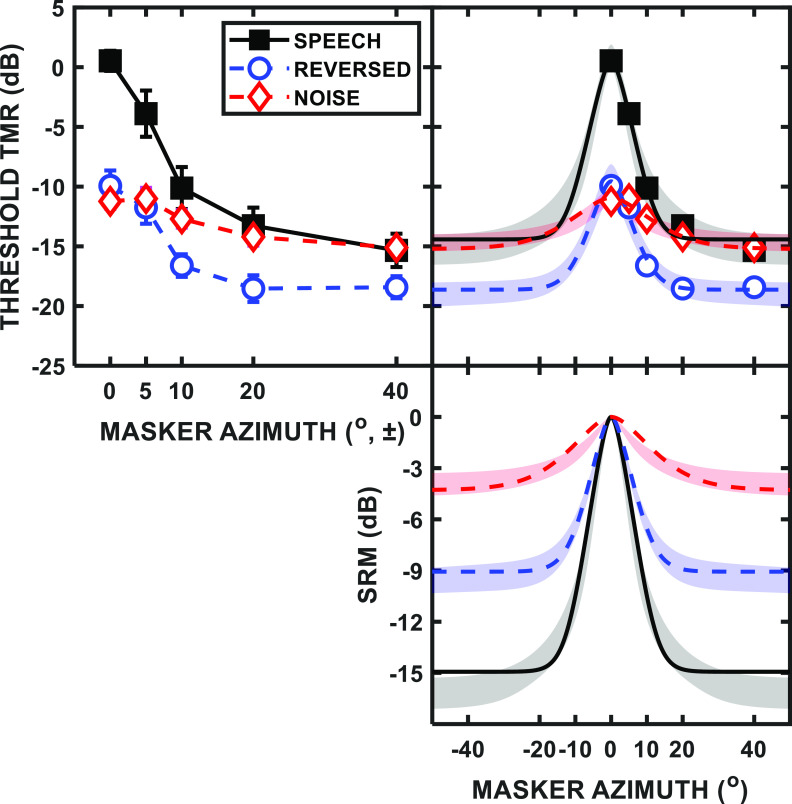
(Color online) Group mean (*N* = 18) results for masked speech identification are plotted in the upper left panel. The values are TMR at threshold plotted as a function of symmetric masker separation from the target. The different functions are for speech (black squares), reversed-speech (blue circles), and noise (red diamonds) maskers. The error bars represent the SEMs. The upper right panel replots the data along with fitted symmetric spatial filters (refer to text) to the group mean results, while the lower right panel aligns the peaks of each spatial filter to show SRM for each masker type. (Note the difference in range of masker azimuth for panels on the right vs upper left.) The shaded regions in the right panels illustrate the ±1 standard error (SE) range of the spatial filter fits across the individual participants.

The measure of performance for masked speech identification was the TMR at threshold (as described above). For each participant and specific condition, the two estimates of threshold TMR were averaged as the final threshold for that condition, and all 18 participant thresholds were averaged for the group mean results. The top-left panel of Fig. 1 plots the group mean thresholds and standard errors of the means (SEMs) for each condition as a function of the spatial separation of the two maskers from the target. The speech masker function (black squares) resembled that of previous research (Marrone *et al.*, 2008; Srinivasan *et al.*, 2016), with colocated SOS thresholds near 0-dB TMR and a substantial improvement in performance observed with greater spatial separation, the SRM effect. A similar trend was also present for the reversed-speech masker (blue circles), albeit with a much lower colocated threshold, while the noise masker (red diamonds) produced a much flatter function as spatial separation of the maskers increased. A 3-by-5 repeated-measures analysis of variance (ANOVA) revealed that both masker type (speech, reversed speech, and noise) [*F*(2,34) = 30.53, *p* < 0.001] and masker separation in azimuth (0, ±5, ±10, ±20, and ±40°) [*F*(4,68) = 97.45, *p* < 0.001] were significant main effects, as was the interaction of the two factors [*F*(8,136) = 16.34, *p* < 0.001].

*Post hoc* paired-samples *t*-tests confirmed that, when colocated, the speech masker produced significantly more masking than either the reversed-speech [*t*(17) = 9.52, *p* < 0.001] or noise maskers [*t*(17) = 16.44, *p* < 0.001]. There was not a significant difference between the reversed-speech and noise thresholds for the colocated conditions [*t*(17) = 1.23, *p* = 0.24]; however, reversed-speech performance was significantly better (lower thresholds) than noise at ±40° separation [*t*(17) = –4.02, *p* < 0.001]. (The Bonferroni-corrected criterion for significance was *α* = 0.013 for this family of *post hoc* tests.) This difference in SRM suggests that, despite both maskers being unintelligible, the reversed-speech masker produced greater IM.

In the same manner as Marrone *et al.* (2008), a best-fitting rounded exponential [roex(*p,r*)] filter function (Patterson *et al.*, 1982; Glasberg and Moore, 1986) was generated for each of the masker types using the matlab functions “*optimset*” and “*fminsearch*,” with the free parameters of *p* (slope/sharpness) and *r* (asymptote), as used in the general equation [Eq. (1)],

y=(1−r)∗(1+p∗x)∗e^(−p∗x)+r, (1)

with *x* being the spatial separation parameter. One side of the filter function is fit to the data (upper right panel of Fig. 1), and the other side is assumed to reflect symmetrically around the peak (Marrone *et al.*, 2008). (The choice of a rounded exponential function was a matter of computational convenience and was not intended to represent any specific physiological mechanism.) These symmetric filters define the spatial tuning/attenuation characteristic and are plotted as lines along with the group mean data points in the upper right panel of Fig. 1 and are replotted in terms of SRM by aligning the curve peaks in the lower right panel. The SRM plot helps to illustrate the similarity in sharpness of tuning (i.e., filter bandwidth) for the speech and reversed-speech maskers for smaller spatial separations, despite the large difference in SRM at the larger separations. The noise curve, however, is both broader and has less of a range than either of the other maskers. The relative influence of IM from each of the maskers could also be inferred from this SRM representation of the results, with the noise masker producing primarily EM and the reversed-speech and speech maskers yielding greater IM.

The normalized values of *p* used by the roex(*p*,*r*) function can be difficult to interpret on their own because they only describe part of the important response of the filter as seen by comparison of the filters derived for speech and reversed-speech maskers in Fig. 1. Filter sharpness or bandwidth also may be expressed in a more intuitive/conventional metric, the separation of the two maskers in degrees needed to reach 3 dB of attenuation relative to the peak (Marrone *et al.*, 2008). Using this metric, the bandwidths of the tuning filters are 7.8° for speech, 10.2° for reversed speech, and 36.2° for noise maskers. Also, the range of the filters—as gauged by the difference between the threshold at the peak (colocated) and the asymptotic SRM—varied by masker type with values of approximately 15, 9, and 4 dB for speech, reversed-speech, and noise, respectively. *Post hoc* paired-samples *t*-tests comparing the bandwidths of the speech and reversed-speech masked spatial filters of the individual participants revealed no significant difference [*t*(17) = –1.33, *p* = 0.20], while there was a difference between the reversed-speech and noise masker bandwidths [*t*(11) = –3.14, *p* = 0.009] (excluding six participants with flat filter shapes for the noise masker, as described below).

**FIG. 2.**
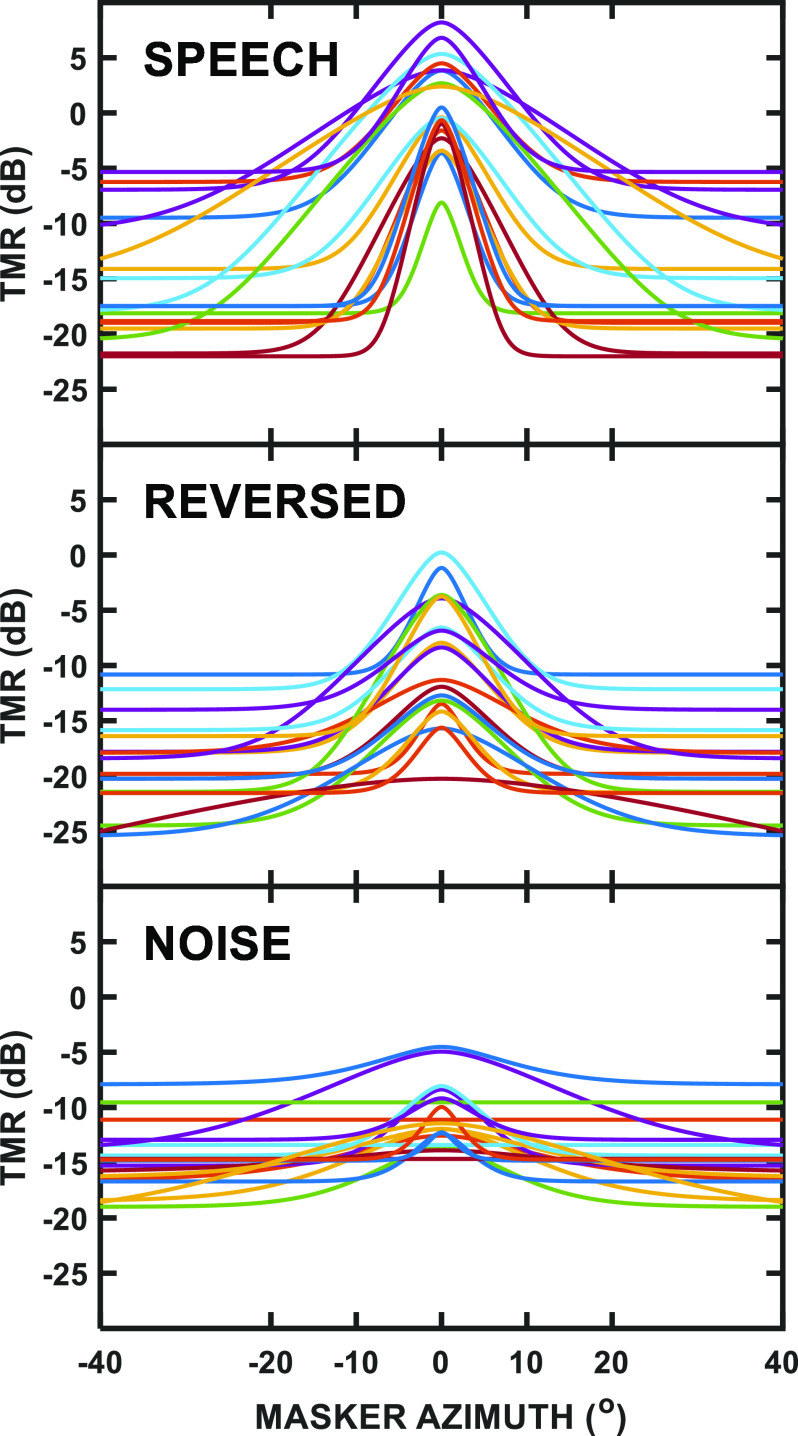
(Color online) Spatial filters for individual participants are plotted for the speech masker (top panel), reversed-speech masker (middle panel), and noise masker (bottom panel), with each line representing a different participant.

The shaded regions in the right-hand panels of Fig. 1 illustrate the ±1 SEMs for the spatial filter fits across individual participants, which are slightly different from the curve fits for the group mean results, yet are in general agreement with those values. The individual participant fits themselves are plotted as separate lines in Fig. 2, with each masker type shown in a different panel. For the speech masker (top panel), there is substantial variability across individuals in terms of curve sharpness, peak (colocated) threshold, and maximum SRM (range) referenced to the peak. Individual differences also are clearly present for the reversed-speech masker (middle panel), while the noise masker (bottom panel) appeared to produce the least variability across participants. For the noise masker, however, the roex(*p,r*) filter was not a better fit to some individual participant's data than simply a constant TMR across spatial separation (in terms of the sum of squares deviations). In these instances, the best-fitting horizontal line was used as the tuning “curve” for that individual.

#### 2. Post hoc observations

**FIG. 3.**
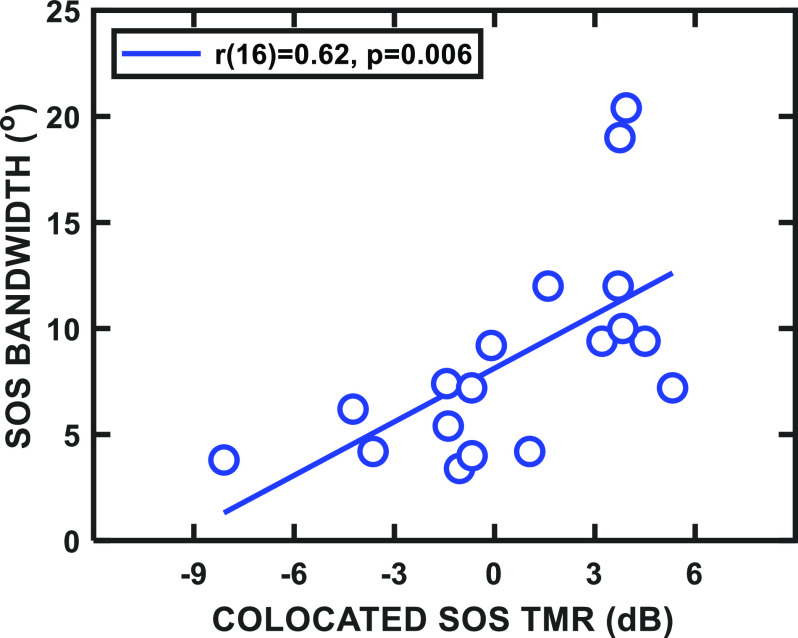
(Color online) Scatter plot showing the bandwidth of the spatial filter for the speech masker as a function of the colocated threshold. The correlation statistics are shown in the key.

Inspection of Fig. 2 revealed that, for the speech masker (top panel), individuals with lower curve peaks also tended to have more sharply tuned spatial filters. This qualitative relationship was not expected based on past work (Marrone *et al.*, 2008), and further *post hoc* analysis seemed warranted. Figure 3 compared the colocated SOS threshold to the bandwidth of the SOS spatial filter, and, in fact, the correlation was significant, indicating that better performance (lower thresholds) for colocated SOS was associated with narrower tuning [*r*(16) = 0.62, *p* = 0.006]. [Supporting results, not presented here, were also found using the slope parameter (*p*) from the roex(*p*,*r*) filter.]

The significant correlation shown in Fig. 3 also implies that the increased ability to separate target and masker talkers based on voice differences—presumably the primary cue for source segregation in the colocated condition—was related to the ability to use small interaural differences to resolve spatially separated sources, which is a prerequisite for sharp spatial tuning. Although other instances have been observed where individual differences in the ability to use source segregation cues were correlated across individual subjects (Kidd *et al.*, 2016; Kidd *et al.*, 2019), and those measures depended on exploiting temporal differences among sounds to achieve perceptual segregation of sources (although the useful interaural differences are on a much briefer time scale than voice differences, such as fundamental frequency), the exact mechanisms and the extent to which they overlap in the different tasks still are not clear. For example, the colocated thresholds and bandwidths were significantly *negatively* correlated in the reversed-speech condition [*r*(16) = –0.61, *p* = 0.008], where presumably the same voice and spatial cues were available for segregating the talkers. The main difference between the two conditions is the intelligibility of the talkers, and it is not clear how that difference influences these trends. For the noise masker (excluding the six participants with flat tuning curves), no significant correlation was found [*r*(10) = –0.13, *p* = 0.68]. (The criterion for significance was *α* = 0.017.) These observations should be examined further given that the subject sample sizes used for the correlations were too small, in our view, to support strong generalizations.

**FIG. 4.**
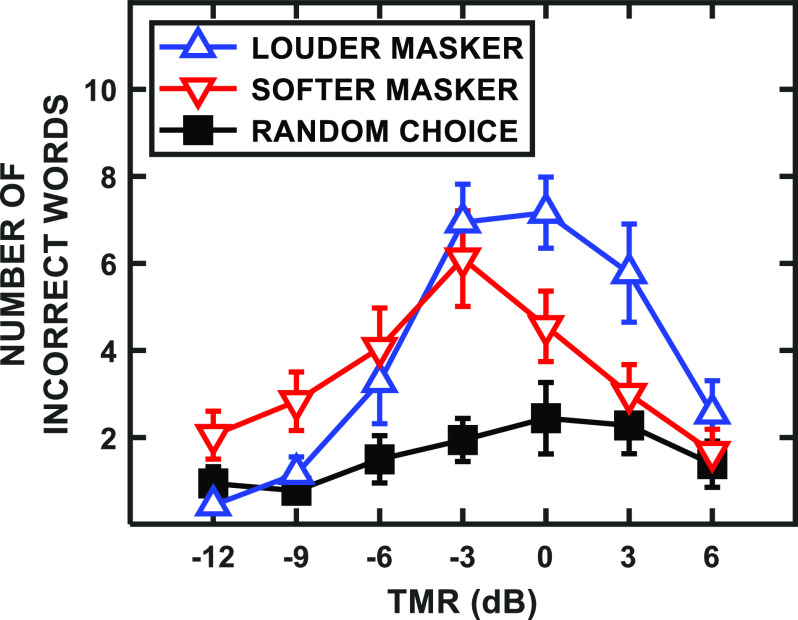
(Color online) Masker confusion functions: the average number of incorrect responses across participants that were either consistent with the louder masker word (blue upward triangles), the softer masker word (red downward triangles), or a random word choice that was not presented during the trial (black squares), as a function TMR. The error bars represent the SEMs. The data plotted include both the 0° and ±5° azimuth speech masker separations.

The randomized level difference imposed between the two maskers on each trial allowed construction of informative masker confusion matrices (Brungart, 2001; Brungart *et al.*, 2001; Kidd *et al.*, 2005; Iyer *et al.*, 2010; Byrne *et al.*, 2022). Figure 4 illustrates the changes that occurred as a function of TMR with regard to the types of errors the participants made. To generate these functions, the errors from both the 0° and ±5° azimuth speech masker separations (highest IM conditions) of each participant were pooled. A 3-by-7 repeated-measures ANOVA revealed that both error type (louder, softer, and random) [*F*(2,34) = 18.14, *p* < 0.001] and TMR (–21 to 6 dB) [*F*(6,102) = 6.88, *p* < 0.001] were significant main effects, as was the interaction of the two factors [*F*(12,204) = 6.75, *p* < 0.001].

At the higher TMR values, a greater number of incorrectly selected words corresponded to the higher-level masker talker (blue upward triangles) on that trial, while for the lower TMRs, the lower-level masker (red downward triangles) was selected more often. *Post hoc* paired-samples *t*-tests comparing the number of confusions with the louder or softer masker confirmed these observations: –9 dB TMR, *t*(17) = –2.73, *p* = 0.007; +3 dB TMR, *t*(17) = 3.05, *p* = 0.004. (The higher-level masker *in isolation* would have ranged from –3 to 0 dB in “TMR” units relative to the composite of both maskers, while the lower-level masker would have ranged from –10 to –3 dB TMR.) Selections of words not presented during the trial (black squares) were generally less frequent. This general pattern of results did not occur for the more spatially separated conditions, where IM was presumably reduced because of the strong segregation cue provided by spatial separation. In those cases, there were roughly equal rates of confusion with the two maskers, as well as a small increase in random choice errors overall.

The pattern present in Fig. 4 was similar to that of Byrne *et al.* (2022), which varied the level differences between a target and two simultaneous, colocated masker talkers, as well as the listener's degree of uncertainty about the target level itself. The results suggested that, when the cue word of a target sentence was noticeably quieter than the maskers, the listeners confused the softer masker with the target more often than the louder, and likely most salient, masker, for which the present results are also consistent. This pattern of results supports the notion of tuning in level (cf. Byrne *et al.*, 2022) in which the focus of attention along the level dimension increases confusions with maskers near the expected level of the target and reduces confusions with masker words remote from the expected target word level.

## IV. EXPERIMENT 2: ANALOGOUS VISUAL SEARCH TASK

The negative correlations between performance on auditory IM tasks and a visual crowding paradigm found by Zhang *et al.* (2021) raised the question as to how general this finding is and whether it would occur for other types of visual (or auditory) tasks. For the present study, a different type of task was chosen to serve as a visual analog for auditory IM. The choice of the visual task was based primarily on its similarity to the auditory task with respect to varying IM. Although devising an exact visual analog of the SOS masking task did not seem feasible (e.g., designing comparable linguistic processing demands), we aimed to at least incorporate the following key elements into the visual task: first, the stimulus/task should vary the degree of uncertainty (or the visual analog of IM) in a controlled manner over an appreciable range, and, second, the physical proximity of the maskers to the target (and thus the “density” near the target location) also must vary. In the auditory task, these elements were implemented by using three different types of maskers—intelligible speech, (unintelligible) time-reversed speech, and noise—and by manipulating the location in azimuth of the masker sources relative to the fixed target.

A prototypical visual search experiment involves a field of visual objects (i.e., visual representations of actual or abstract characters/images), which the participant must inspect while attempting to find a specific cued target object [refer to Wolfe (2014) for a review]. By varying the properties of the target and distractors in a controlled manner, the expected search time (time to identification of the cued target) also may be varied systematically. As with auditory sources, the more dissimilar the distractors (which we also refer to as maskers but make a distinction between “distractors” and “maskers” in the experiment described below) are from the target (e.g., differences in color or size), the better the expected performance (faster the search times). In contrast, slower search/response times occur when the distractors possess features similar to the target and/or must be distinguished from the target based on multiple feature dimensions (“feature” vs “conjunction” searches; Treisman and Gelade, 1980; McElree and Carrasco, 1999). Manipulating the structural similarity of target and distractor objects (Wolfe and DiMase, 2003) could be seen as analogous to varying the strength of IM with the different auditory maskers used in experiment 1 because target-masker similarity was varied across the three masker types.

Furthermore, within the field of vision encompassing the likely location of the target object—the search region—the proximity of individual maskers to the target as well as the overall density of the display (i.e., the number of objects within the search region) would be expected to affect the difficulty of the search task. As with the spatial SOS masking task described in experiment 1, where increasing the spatial separation of the speech maskers from the target talker resulted in a decrease in the “density” of the target location (i.e., a decrease in the number of talkers at the known location of the target) as well as the proximity of the target to each individual masker. However, the differences in individual performance in visual search are likely to result from the efficiency with which the search task is solved (search strategy, feature conjunction/integration, etc.) rather than through different attenuation characteristics of a spatial filter as postulated for the SOS task.

### A. Methods

The visual search experiment consisted of a “cloud” of eight black objects, which were always placed within a 9 row by 5 column grid (not visible to participant) at the center of an all-white computer display. The objects in this cloud were always identical except for their angular orientation. Among these eight identical objects, the “target” had a unique angular orientation, which was designated to the participant by a cue before each set of trials. The objects of the cloud that were at uncued orientations but still were exact matches to the target are henceforth called *distractors* and are distinct from the “masker” objects described below. The designated target was always a capital “T” with an angular rotation chosen at random for each set of trials from the set of 45° to 315°, with 45° increments. (The target was never at 0° rotation; never a right-side-up “T.”) The distractor objects in the target cloud used each of the other six possible rotations, as well as a 0° angular rotation. The participant began each set of trials self-paced and could take as much time as needed to memorize the cued orientation before beginning. The task of the participant was to find and click on (using the computer mouse with a crosshairs cursor) the target as fast as possible.

In addition to the *distractors* described above, the *maskers* for the visual search task always comprised two clouds, each made up of 16 objects. These two “masker clouds” were simultaneously displayed with the target/distractor cloud and were either colocated within the 9 × 5 grid of the target cloud or were spatially, and symmetrically, shifted left and right by a certain number of grid columns, as specified by different conditions. The column offset was either 0 (entirely colocated), ±1, ±3, ±5, or ±7 columns, with the final two offsets having no overlap with the target/distractor cloud region. In addition, all target/distractor and masker object placement within their respective clouds was randomized for each trial, with the limitation being that only one object could fill each grid space (i.e., there was no overlap of any objects). This resulted in a very dense colocated condition with only five grid spaces open within the 9 × 5 (possible target location) region.

**FIG. 5.**
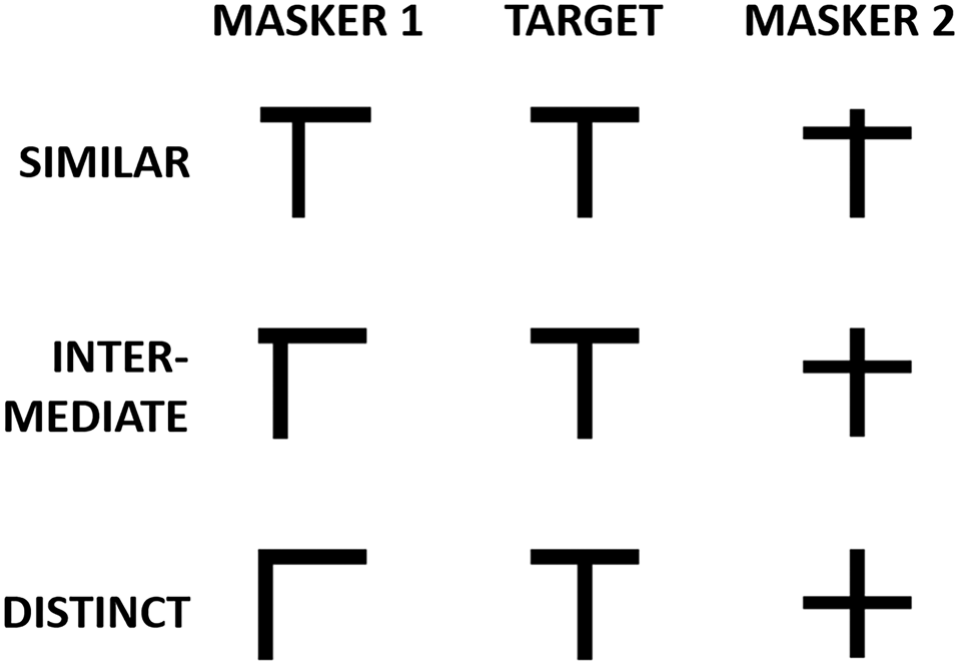
The types of maskers used for the similar (top row), intermediate (middle row), and distinct (bottom row) masker conditions. The target is shown in the center column for comparison (although depicted at 0° rotation, which was not allowed in the experiment). On each trial, one masker cloud was formed using the “masker 1” set, while the second masker cloud was formed using the “masker 2” set.

The maskers were derived from the target object in a manner that has been used in previous studies of visual search (Wolfe and DiMase, 2003) and are depicted in Fig. 5. For the “similar” masker condition (Fig. 5, top row), the two maskers were formed from only a small shift in the two perpendicular lines that made up the target “T.” The “intermediate” and “distinct” masker conditions (middle and bottom rows, respectively) shifted the two lines to greater extents, resulting in objects similar to “<” (or “L”) and “+” symbols in the distinct masker condition. Each of the two masker options (from the “masker 1” and “masker 2” columns in Fig. 5) were used for separate masker clouds, each cloud with its own 9 × 5 grid. To create the 16 objects for each masker cloud, two instances of each masker object were presented from the eight possible 45° spaced rotations. For spatially separated conditions, which of the two masker objects (“1” or “2”) was used for the left or right masker clouds was randomized on each trial.

**FIG. 6.**
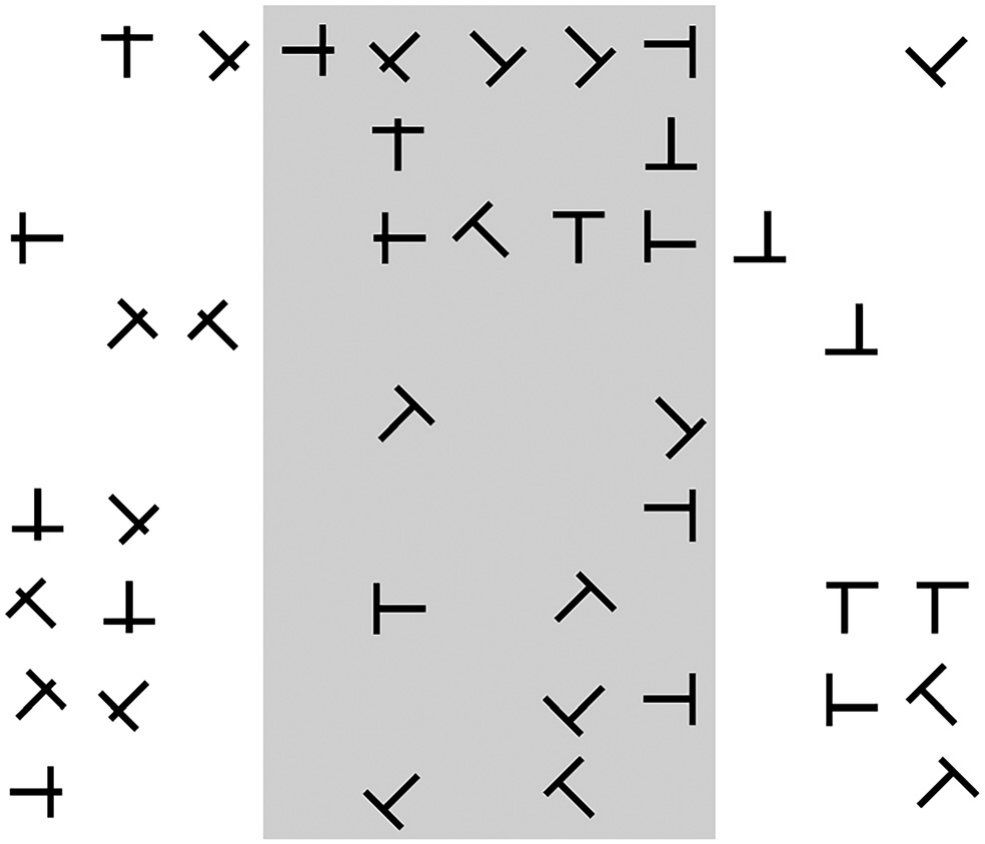
An example (practice) trial of the visual search task for the similar masker condition, with a three-column offset for the maskers. The gray shaded region, which was only included during initial practice, highlights the ideal search region where the target could be located.

The overall search task was, therefore, a conjunctive visual search (Treisman and Gelade, 1980), because the participant must find the object that is both the correct structural form *and* cued orientation, with both object size and color held constant. Each participant was shown 5–10 sample trials (always using the similar masker condition) to familiarize them with the task prior to formal data collection. An example practice trial is shown in Fig. 6, including a gray shaded region that was utilized only for these practice trials. This shaded region explicitly showed the participant the area within which the target object could be located (the ideal search region). It was also shown during the presentation of the cue prior to each set of trials as a reminder. (This was akin to the explicit instructions given in experiment 1 that the target voice would always sound directly forward.) This gray region was not included during the presentation of objects during the experimental blocks of trials.

Experiment 2 was implemented using the same computers and software and within the same sound-attenuating chambers as experiment 1. For all participants, a 22-in. computer monitor with a 1920 × 1080 pixel resolution was used, with 43 × 43 pixel dimensions for all target and masker objects. (The target cue before each set of trials was presented with a 150 × 150 pixel size.) The invisible grid for possible object placement used 75 × 75 pixel cells, with the objects centered both horizontally and vertically within a cell. The viewing distance of individual participants was not standardized or measured.

Experiment 2 consisted of six blocks, which included a set of (at least) ten trials for each of the three masker types and five masker separations, grouped separately. The order of the condition sets within each block was randomized. Correct answer feedback was given after each trial (circling the correct target object location), with an incorrect answer defined as any response more than 50% outside the dimensions of the target object's grid space. This buffer was used to account for imperfect/lazy mouse clicking. Ten correct responses were required to terminate each set. As with experiment 1, participants ran the experiment self-paced over the course of multiple sessions, along with the task of experiment 1. The total time required to complete experiment 2 was approximately 1.5 h, not including participant break periods.

### B. Results and discussion

**FIG. 7.**
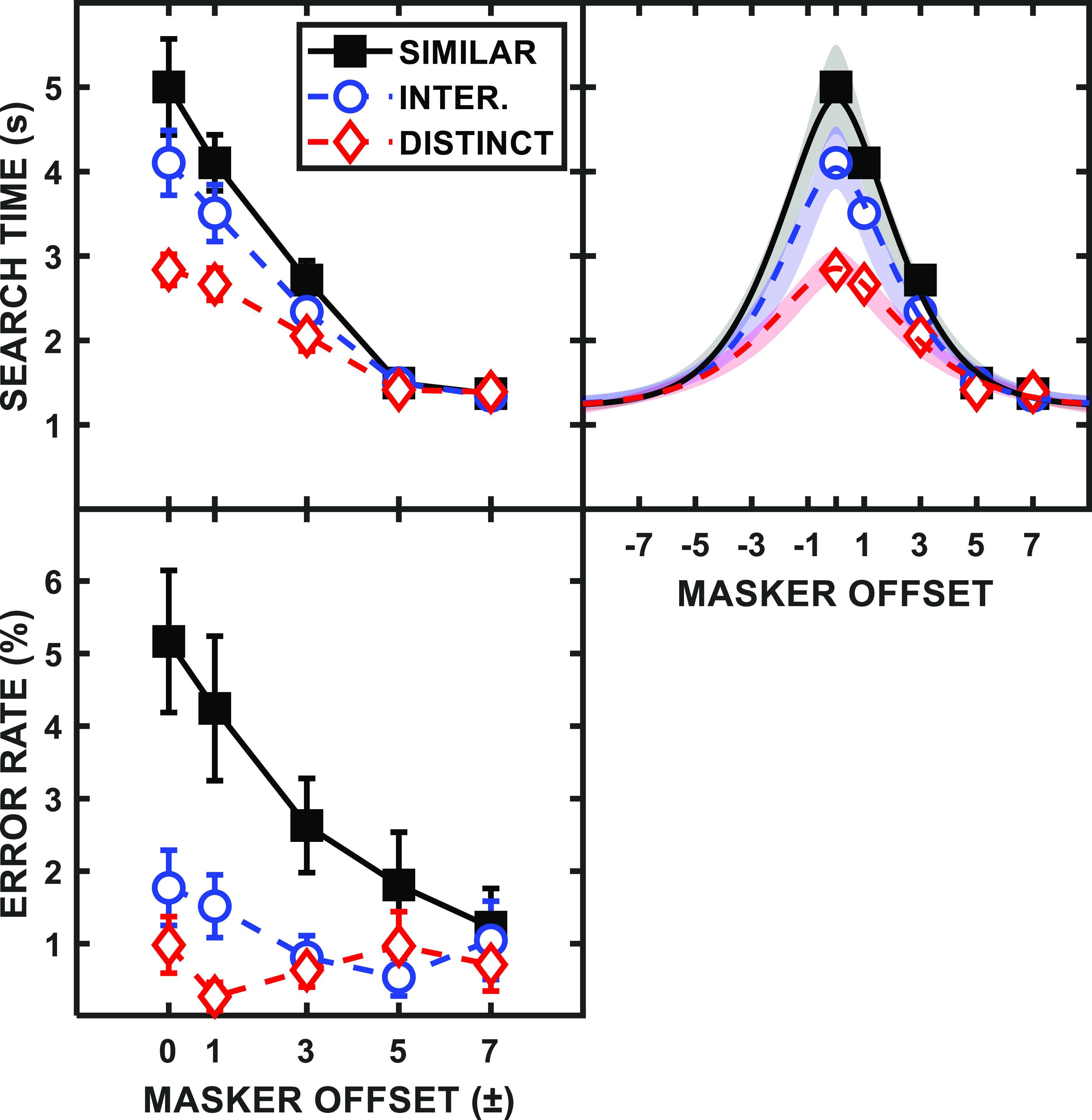
(Color online) Group mean (*N* = 18) search times for visual search are plotted in the upper left panel as a function of the masker-cloud offset (columns) from center. (Because the number of masker objects in the ideal search region decreased with masker-cloud offset, increasing masker offsets also can be interpreted in terms of decreasing set size.) The functions are for the different masker types: similar (black squares), intermediate (blue circles), and distinct (red diamonds). Error bars represent the SEMs. The upper right panel plots the search time data fit with symmetric curves. The shaded regions illustrate the ±1 SE range of the visual masker function fits for the individual participants. The error rates for the different conditions are shown in the lower left panel.

For experiment 2, the measure of visual search performance for each participant was the median value of the 60 total (correct response) search times for each specific condition, with error trials discarded for this measure. Performance of all 18 participants was then averaged for the group mean results, which are plotted in Fig. 7, in a manner similar to that for experiment 1 (cf. Fig. 1). The top left panel of Fig. 7 shows the mean search times for each condition as a function of the (symmetric) masker-cloud offset from the target cloud in terms of grid columns. Note that, in this design, increasing the masker-cloud offset reduced both the proximity of individual maskers to the target *and* the density of the search region. As such, larger masker-cloud offsets can be interpreted in terms of smaller set sizes within the search region (i.e., the area identified prior to a block of trials as the region where the target would occur). All 40 objects were located within this region with a masker offset of 0, while the number decreased to 34 and 21 (on average) for offsets of ±1 and ±3, respectively, and only the eight target cloud objects were located in this region for the two largest masker offsets.

Search time decreased for all three masker types as the maskers were increasingly spatially separated from the target (i.e., as proximity and density decreased). Qualitatively, then, this finding is similar to the effect of spatial separation in experiment 1, where, under high-IM conditions, decreasing target-masker proximity (and, by analogy, the source density of the target location) was associated with improved performance. The similar masker (black squares) produced the longest search times when colocated [M = 5.00, SE = 0.57], while the distinct masker (red diamonds) had the fastest search times (M = 2.84, SE = 0.18). The colocated intermediate masker (blue circles) fell between the two extremes (M = 4.10, SE = 0.38), but all three masker functions come together at the two largest masker offsets (the two offsets with no target-masker cloud overlap).

A 3-by-5 repeated-measures ANOVA revealed that both masker type (similar, intermediate, and distinct) [*F*(2,34) = 43.53, *p* < 0.001] and masker offset (0, ±1, ±3, ±5, ±7 columns) [*F*(4,68) = 78.07, *p* < 0.001] were significant main effects, as was the interaction of the two factors [*F*(8,136) = 12.80, *p* < 0.001]. While the three different masker functions clearly aligned for the largest offsets, *post hoc* paired-samples *t*-tests confirmed that, with no target-masker offset, the similar masker produced significantly more masking than the intermediate masker [*t*(17) = 2.25, *p* = 0.019], while the intermediate masker produced significantly more masking than the distinct masker [*t*(17) = 5.02, *p* < 0.001].

The error rates, although relatively small, also were influenced by the degree of target-masker similarity (amount of IM), as shown in the lower left panel of Fig. 7. Considerably more incorrect responses occurred for the similar masker than for either of the other two masker types, at least for the closely spaced masker offsets, which included overlaps with the target cloud. A 3-by-5 repeated-measures ANOVA on the error rates revealed that there were significant main effects of masker type [*F*(2,34) = 29.05, *p* < 0.001] and masker offset [*F*(4,68) = 6.11, *p* < 0.001], as well as a significant interaction [*F*(8,136) = 4.45, *p* < 0.001]. *Post hoc* tests confirmed that, with no masker offset, the similar masker produced significantly more errors than the intermediate masker [*t*(17) = 3.45, *p* = 0.002]; however, there was no significant difference in the number of errors observed between the intermediate and distinct maskers [*t*(17) = 1.64, *p* = 0.059].

Qualitatively, the pattern of group mean results in the upper left panel of Fig. 7 resembles the pattern of group mean results in the upper left panel of Fig. 1. In both cases, performance improved with spatial separation of the maskers (i.e., with decreasing target-masker proximity and object density of the ideal search region); for the speech intelligibility task, the metric that varied with spatial separation was TMR at threshold, while for the visual search task, the metric was the time needed to correctly identify the target. Furthermore, in both cases, the poorest performance occurred for the most similar masker without the benefit of masker spatial separation, while the performance difference between masker type declined as spatial separation increased (i.e., as fewer confusable maskers fell within the spatial region known to contain the target).

The similarities between the patterns of results across modalities suggested that applying the same curve-fitting procedure used in experiment 1 might be useful for representing the data in experiment 2, that is, fitting a similar filter-like pattern to the search time vs masker separation data and estimating a “bandwidth” and “range.” It should be emphasized that while the results from the curve-fitting to the search time data yield patterns resembling the auditory spatial filters, we are not claiming that the underlying mechanism is the same (i.e., that the fitted curves represent an attentional filter tuned along the spatial dimension, as was the case in experiment 1) and refer to the curves displaying the visual search data as “visual masker functions.” Indeed, we are not attributing the pattern of search times to any specific underlying physiological mechanism. However, this summary of the visual data facilitates the comparison—allows similar metrics to be derived—across modalities of the effects of different IM levels on both individual subject and group mean performance.

**FIG. 8.**
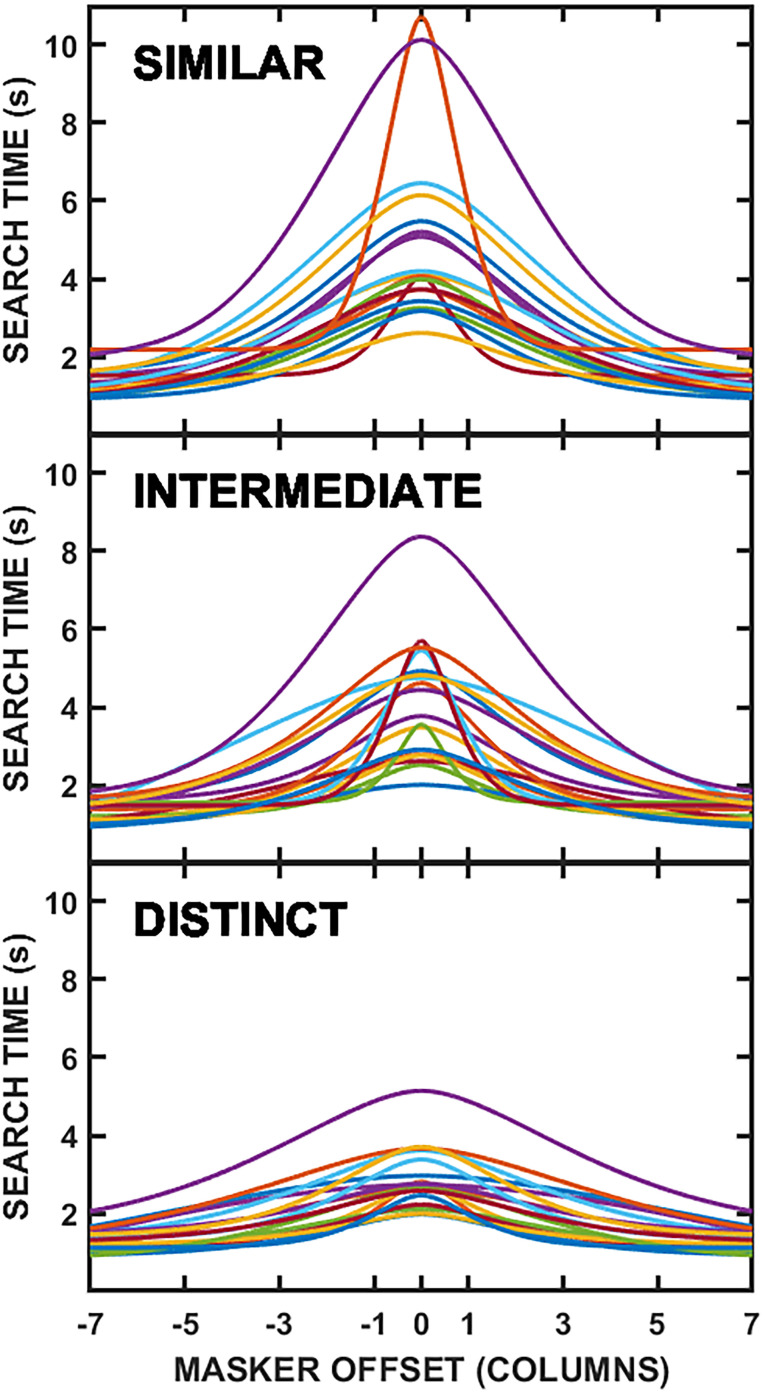
(Color online) Visual masker functions, illustrating the relationship between search time and masker-cloud offset for each individual participant for the visual search task, are plotted for the similar masker (top panel), intermediate masker (middle panel), and distinct masker (bottom panel), with each line being a different participant.

Thus, in the same general manner as in experiment 1, best-fitting roex(*p*,*r*) curves were generated for each of the visual masker functions and were plotted as lines in the upper right panel of Fig. 7. As in Fig. 1, the shaded regions show the ±1 SEM ranges for the visual masker functions (curve fits) across individual participants, with the individual participant fits themselves plotted as separate lines in Fig. 8. Although there were only small individual differences in search time for the largest masker offset (only a 1.26-s range across all three maskers and participants), there were considerable ranges in colocated performance for the similar and intermediate maskers (8.11 and 6.37 s ranges, respectively). This trend for greater individual differences in the most similar masking condition parallels the results from the speech intelligibility task as well as the other comparisons noted above.

## V. CORRELATES OF SPEECH IDENTIFICATION PERFORMANCE

One goal of the current study was to determine whether performance on a visual IM task and/or a few other selected metrics would predict individual differences in performance on an auditory IM task. To facilitate these comparisons, which involve different scales, the data obtained from the speech intelligibility, visual search, and the RAPM tests were converted to *z*-scores. For speech intelligibility, the average TMR at threshold across all conditions of experiment 1 was computed for each participant. These values were then transformed into *z*-scores (with the sign flipped such that higher values indicated better performance), thus, creating a value considered to reflect overall performance for masked speech identification. The same process was used for the average visual search times across conditions of experiment 2, and for consistency, the RAPM scores were also transformed into *z*-score units (cf. Fullgrabe *et al.*, 2015). Musical training was also used as a predictor variable but, because it was a self-report index and not a performance metric, was not transformed to *z*-scores.

**FIG. 9.**
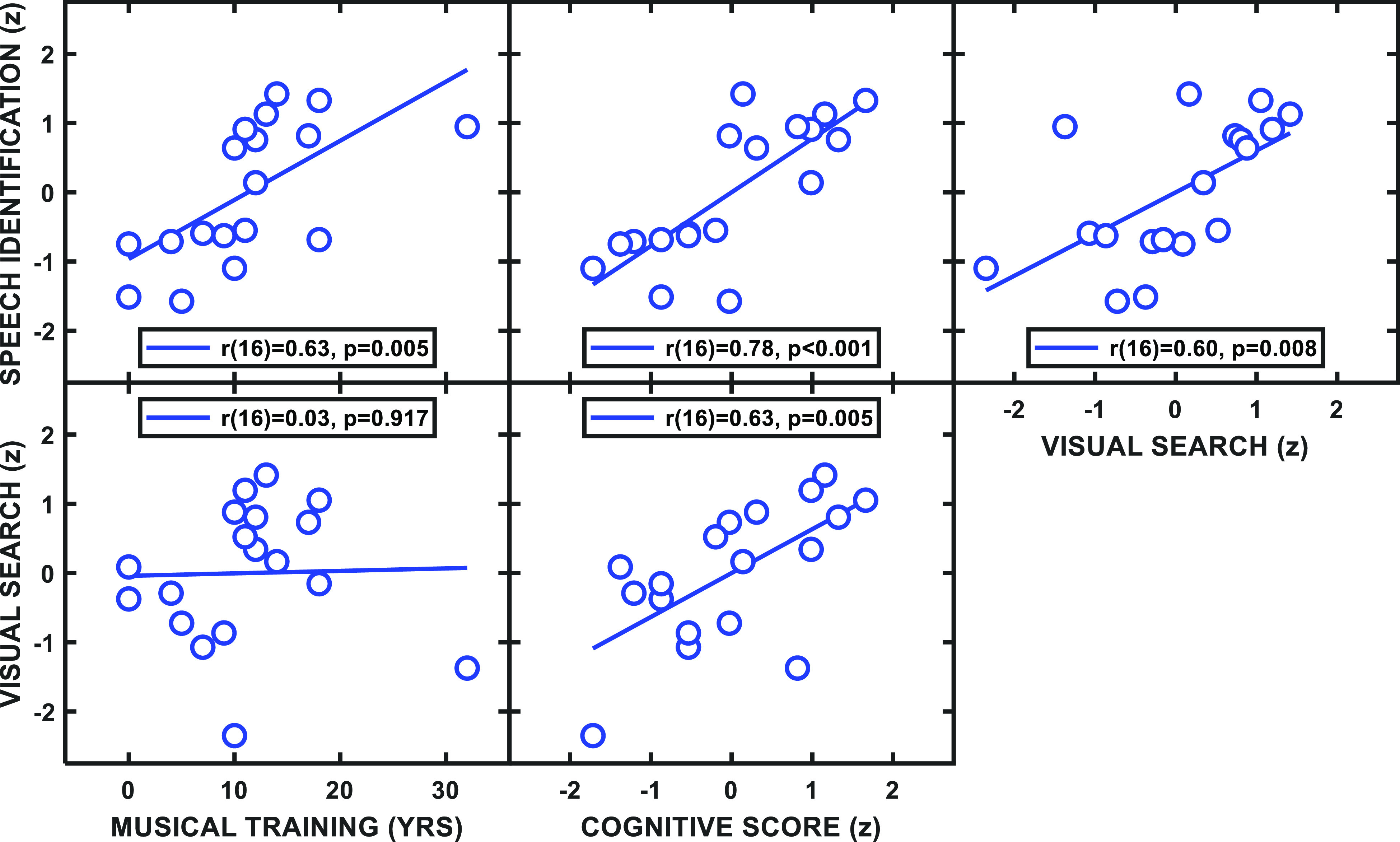
(Color online) The upper row shows scatter plots and correlations between overall speech identification performance (in *z*-score units, see text) from experiment 1 and three different metrics: years of musical training (left panel), cognitive score (RAPM; middle panel), and overall visual search performance (right panel) from experiment 2. Variables have been transformed such that higher values indicate better performance. The correlation statistics for each panel are shown in the key. The lower panels are similar plots for performance on the visual search task as a function of years of musical training and cognitive score.

Plots of the correlations between overall speech identification performance and these hypothesized predictor variables are shown in the upper panels of Fig. 9. There were significant (positive) correlations between speech identification performance and the number of years of musical training [*r*(16) = 0.63, *p* = 0.005, top-left panel], cognitive score [*r*(16) = 0.78, *p* < 0.001, top-middle panel], and visual search task performance [*r*(16) = 0.60, *p* = 0.008, top-right panel]. Cognitive task performance also was correlated with performance on the visual search task [*r*(16) = 0.63, *p* = 0.005, bottom-middle panel], but years of musical training was not [*r*(16) = 0.03, *p* = 0.92, bottom-left panel]. (The criterion for significance was *α* = 0.01.) The lack of significance between visual search performance and the years of musical training should be interpreted cautiously. All subjects were included in the present analysis, while the subject with the most years of musical training performed particularly poorly on the visual task, and those data exerted a strong influence on the correlation; thus, further investigation of this relationship with a larger sample size is warranted.

A multiple regression model indicated that auditory performance was significantly predicted from the combination of years of musical training, cognitive score, and visual performance [*F*(3,14) = 15.29, *p* < 0.001, *r*^2^ = 0.77]. Musical training and visual performance added significantly to the prediction (*t* = 2.94, *p* = 0.01 and *t* = 2.33, *p* = 0.04, respectively), while the cognitive score did not (*t* = 1.08, *p* = 0.30). Despite auditory performance and cognitive score having the strongest correlation in Fig. 9 (top-middle panel), there is also a rather substantial spread of overall speech identification performance for cognitive scores near the midline, which leads to this contradiction. It also was noted that two apparently distinct groups emerged in the comparison of auditory and RAPM performance. One of these groups (categorized informally by our subjective scrutiny) had higher performance on both tasks, while the other group was noticeably lower for both. Given this observation, a *post hoc* comparison of these two groups (which coincidentally had equal numbers of subjects) was performed to determine whether this informal observation rose to the level of statistical significance.

**FIG. 10.**
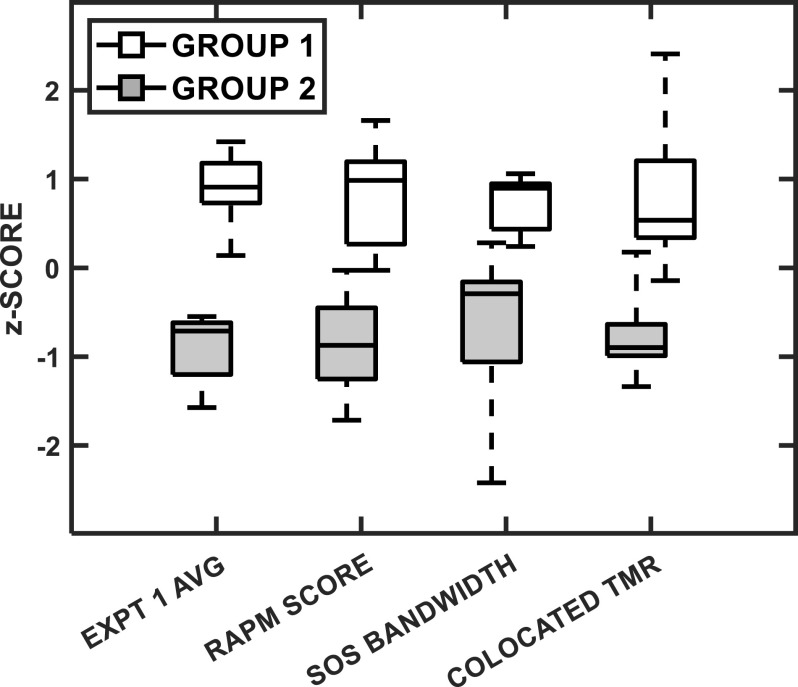
Box plots comparing performance for two participant subgroups (groups were formed *post hoc* on the basis of performance; see text) are presented after *z*-score transformations. Each pair of box plots is for a different metric, as indicated by the *x* axis labels: (1) average performance across all conditions in experiment 1, (2) cognitive (RAPM) score, (3) bandwidth of the SOS spatial filter, and (4) the colocated SOS threshold. In all cases, higher values indicate better performance. The box ranges indicate the 25th and 75th percentiles, with the center lines being the median values. The dashed bars extend to the full range of the data for each group.

Figure 10 presents (*z*-score transformed) box plots for each group, which were divided evenly between the nine participants with the highest overall speech identification performance (group 1) and the nine participants with the lowest performance (group 2), in terms of four variables chosen of particular interest. Independent-samples *t*-tests revealed significant differences between groups for both overall speech identification performance [*t*(16) = 9.75, *p* < 0.001] and RAPM cognitive score [*t*(16) = 6.18, *p* < 0.001]. The other two variables chosen were the bandwidth of the SOS spatial filter and the colocated SOS threshold, two measures of a participant's susceptibility to IM that were found to be correlated with each other in experiment 1 (Fig. 3). For both additional measures, the differences between groups were also significant [SOS bandwidth: *t*(16) = 4.34, *p* = 0.001; colocated TMR: *t*(16) = 5.27, *p* < 0.001]. (The criterion for significance was *α* = 0.0125.) The Cohen's *d* effect sizes for all four group comparisons were large (*d* > 0.8), with values of 4.6, 2.9, 2.0, and 2.5, respectively.

These trends suggest a distinction between higher performing participants with lower overall masked speech identification thresholds, sharper SOS spatial release of masking, less IM present for colocated SOS, and higher cognitive reasoning scores. Although this comparison is intriguing, further work with a larger subject pool is needed to determine whether in fact there is a bimodal element to the distribution of performance or, rather more likely, the subjects sampled here happened to fall into two somewhat distinct groups. The tendency for individuals to exhibit correlated performance on multiple metrics, though, seems to be genuine and is broadly consistent with the informal classification of “good” vs “poor” performing listeners (cf. Kidd *et al.*, 2016).

## VI. GENERAL DISCUSSION

In the present study, a group of adult subjects participated in auditory and visual tasks that were intended to measure similar influences on task performance under analogous IM conditions. In each sensory modality, two factors were varied by the choice of stimuli and experimental design: (1) the degree of IM (or an analog of IM in vision) and (2) the physical (spatial) proximity of individual maskers to the target, along with the source density of the target region (the known location of the target in the auditory task and the ideal search region in the visual task). These factors resulted in significant differences in performance when considered in terms of group means. Overall performance (across all conditions, including both low- and high-IM maskers) was significantly correlated across individuals for speech identification accuracy and visual search time to correct identification. This broad-brushstroke finding suggests that there is at least some common mechanism that is shared between modalities when solving IM tasks.

For both speech identification accuracy and visual search time, performance was modulated by the degree of target-masker similarity. If it is assumed that target-masker similarity causes observer uncertainty (i.e., the more similar the stimuli, the greater the uncertainty about which is the target), and uncertainty is the basis for IM (Watson, 1987; Kidd *et al.*, 2002; Durlach *et al.*, 2003a; Durlach *et al.*, 2003b; Kidd *et al.*, 2008b), then performance on both tasks declined as the degree of auditory IM and its visual analog increased. Specifically, the performance metrics were poorer (higher TMRs were observed for the auditory task for the highest IM masker; longer search times were found on the visual task for the masker most similar to the target), more errors/confusions were observed with the most similar masker (with the smallest spatial separations), and greater intersubject variability was apparent in both tasks for the most similar masker.

For both the auditory and visual IM tasks, spatial “filters” were fit to the results as a means to describe the effects of proximity and density under different levels of IM. There were clear similarities between the patterns of results for the two tasks. Masking was greatest in the auditory task, and search times were greatest in the visual task, when proximity/density was high, and both masking and search times decreased with decreasing proximity/density. Less tuning (both in terms of bandwidth and depth of tuned responses) was found for the least similar (lowest IM) maskers in both modalities. Thus, qualitatively at least, the stimulus manipulations in the different modalities yielded similar “tuned” patterns that were similarly modulated by IM.

Again, it should be noted that we do not think that the tuned patterns for the two modalities reflect the same attentional mechanism. That is, the auditory filters can be interpreted as auditory spatial attentional filters centered on the target location (Arbogast and Kidd, 2000; Marrone *et al.*, 2008), with sharper tuning/greater depth of tuned responses likely reflecting more focused spatial attention. The visual masker functions, by contrast, can be interpreted as search-time by set-size functions that likely reflect the combined influence of multiple abilities/factors (e.g., the ability to focus attention on individual objects in the search array, the ability to engage/disengage attention on individual objects, the ability to efficiently process the object at the focus of attention, etc.).

The two tasks also implemented target variability/uncertainty in different ways. The speech target was presented at a fixed location, which greatly reduced target uncertainty for the spatially separated conditions. By comparison, the visual target had an uncertain location within a fixed ideal search area. However, in some respects, the target varied more for the auditory task because the target talker was randomized on every trial, while the visual target was cued and remained fixed in orientation for an entire block. Nevertheless, the fact that both the auditory and visual tasks yielded similar “tuned” patterns—performance improving with decreasing proximity/density and degrading more rapidly under high-IM conditions—suggests at least some overlap of the mechanisms that contributed to performance in the two tasks.

**FIG. 11.**
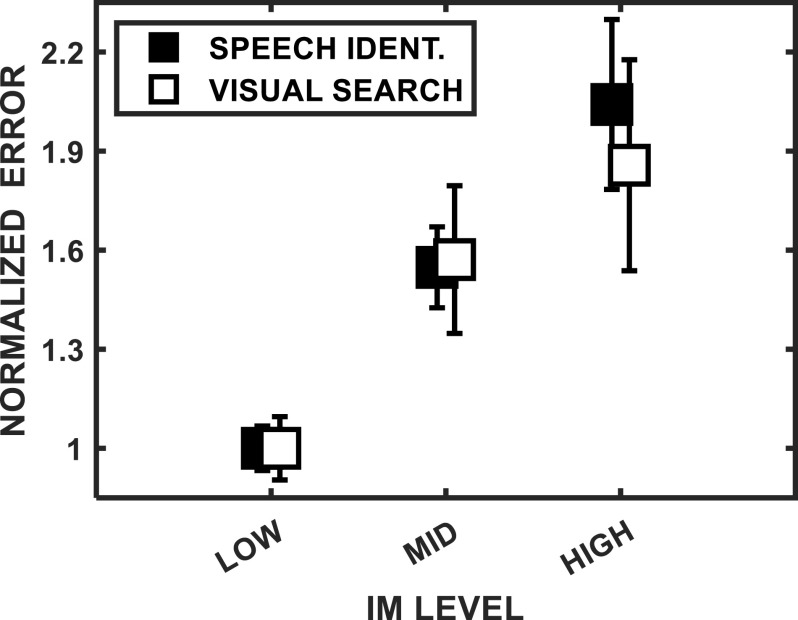
Normalized inter-subject SEs (averaged across all masker spatial separations) as a function of the expected strength of IM. For the auditory (A) and visual (V) tasks, the IM conditions were as follows: “low,” noise (A) and distinct (V); “mid,” reversed (A) and intermediate (V); and “high,” speech (A) and similar (V) (see Secs. III A 1 and IV A for further descriptions). The values were normalized by dividing the mean SEs for the mid and high conditions by the mean SE for the low IM condition [i.e., these are ratios of SEs relative to those obtained in the noise (A) and distinct (V) conditions]. The error bars are the SEMs, the variability of SEs across all masker spatial separations.

Across the subject pool, large individual differences were found for both auditory and visual tasks that varied according to the strength of IM. As may be seen in Fig. 11, the variability in performance across subjects increased as the degree of IM increased. This general finding is consistent with a large body of previous research in hearing (Kidd *et al.*, 2008b), and the generalization to visual IM also was expected. In one sense, this result is a validation of the intended effect of the variation in IM incorporated into the experimental design.

The present results, particularly when the findings from Zhang *et al.* (2021) of a *negative* correlation between performance on (high-IM) auditory and visual tasks are taken into account, suggest that a large measure of caution is needed when interpreting comparisons of the results of auditory and visual experiments. Broadly, our findings imply that there is indeed evidence of a common component that influences overall performance in solving selective attention tasks with different levels of IM, but the ability to make use of the specific features and properties distinguishing target and masker stimuli is likely modality-specific. This raises the question of whether higher-level abilities, such as exploitation of *a priori* probability and expectation (cf. Kidd and Colburn, 2017), also would exhibit modality-specific processing that must be taken into account.

The visual search task used here and the visual crowding task of Zhang *et al.* (2021) likely measure two very different aspects of IM in the visual domain. Limitations on performance in visual crowding involve the inability of the viewer to distinguish a target object in peripheral (yet attentionally focused) vision from nearby distractor objects. When the target and distractors are too close together, they are typically perceived as an “unidentifiable jumble” (Pelli, 2008), a description that suggests a process distinct from auditory IM. Even when competing talkers are equal in level and colocated, a listener often can still identify a high proportion of the words that were present (Kidd *et al.*, 2016), even if those words were from a masker rather than the target talker; that is, competing speech may be incoherent, but individual words are usually not “unidentifiable.”

An underlying rationale for the visual search task used here as an analog to SOS masking was that IM for speech identification—at least in the colocated presentation—is akin to conjunction searches in the visual domain. As a listener hears each talker within a mixture of multiple simultaneous talkers, they must determine whether the characteristics of the attended speech source (e.g., vocal tract resonances, intonation, and loudness) are consistent with the cues that define the target talker and, thus, whether the words emanating from that source should be stored in memory; if not, the utterance should be ignored and not stored for recall. In the visual search domain, this process is somewhat similar to a conjunction search of each object present to determine whether it matches the cued target object on all relevant properties (serial search; Treisman and Gelade, 1980). If the masker objects are distinct enough from the target, e.g., a different color, the task becomes a feature (or parallel) search in which all objects not of the target color can be ignored, resulting in an easier, faster search. Thus, to that extent, a feature search in a multi-object visual field may be similar to distinguishing the features designating a target voice among dissimilar competitors (e.g., when masker talkers are of the opposite gender from the target, SOS performance often is greatly improved; Kidd *et al.*, 2016).

In the present visual search task, as the masker clouds were spatially separated from the target cloud, the object density within the search region decreased, reducing the number of objects that needed to be searched. For the masked speech identification task, the density of sources (and, thus, the array of features that must be searched to determine the target) also varied with proximity of the maskers if one assumes that an auditory spatial filter is centered on the target location. Spatial separation of maskers improved performance despite only small changes in the amount of EM that is occurring (cf. “better-ear glimpsing”; Best *et al.*, 2015; Best *et al.*, 2017). Spatially separated maskers can be more easily ignored because they fall outside of the spatial filter, thereby reducing the speech “density” to only the voice that emanates from the attended spatial location. Therefore, in that regard, the density of the spatial field that must be searched for relevant features varies for both the visual search task and the auditory speech identification task in a similar way.

Although the perceptual and cognitive mechanisms involved in solving auditory and visual tasks differ in important ways (partly reflected in the different dependent variables used of accuracy and search time, respectively), the extent to which specific processes dominate depends in both cases on the stimuli used. For the visual task, successful search with high-IM stimuli presumably relied more heavily on top-down processes than for low-IM conditions, while only the largest target-masker offsets would have allowed for more bottom-up processing, given the increase in target salience within the ideal search region [refer to Patel and Sathian (2000) and Wolfe (2014)]. For the auditory task, the salience of the target speech not only varied across conditions but also was affected by the trial-by-trial TMR. Identification of target speech near or just below threshold can require considerable top-down processing as the listener attempts to identify an incomplete word by filling in the time-frequency elements that are missing due to EM (Brungart *et al.*, 2006; Zekveld *et al.*, 2006). For SOS performance, which is known to depend on certain cognitive abilities as discussed elsewhere in this article, initial segregation of talkers likely also depends on bottom-up processing, when the listener must first isolate the vocal characteristics of the target talker distinct from the maskers (Brungart *et al.*, 2001).

It was noteworthy that the better performing subjects on either task had higher scores on the cognitive test, and given the above considerations, the extent to which cognitive ability might be predicted by either modality also was of interest. Furthermore, *post hoc* multiple regression models indicated that RAPM scores were significantly predicted from the different levels of IM for each modality. However, for the auditory task [*F*(3,14) = 12.06, *p* < 0.001, *r*^2^ = 0.72], only the high-IM, SOS masker added significantly to the prediction (*t* = –3.88, *p* = 0.002), while performance with reversed-speech or noise maskers did not (at an *α* < 0.05 criterion). The same was true for the visual task [*F*(3,14) = 7.32, *p* = 0.003, *r*^2^ = 0.61], with only the similar masker adding significantly to the prediction of cognitive score (*t* = 2.66, *p* = 0.02), while performance with the intermediate and distinct maskers did not significantly contribute. Therefore, regardless of modality, cognitive performance was well predicted by a high-IM task.

The positive correlation between auditory performance and musical training was also expected based on past findings. Swaminathan *et al.* (2015) and Clayton *et al.* (2016) both reported that musicians as a group achieved better performance on similar SOS tasks, especially when the maskers were spatially separated from the target. Clayton *et al.* (2016) also reported that the musician group performed better on the DSB cognitive test than did nonmusicians. The finding that performance on a cognitive measure was better in the musician group implies that there may be cognitive abilities that underlie the superior performance musicians exhibit in solving the SOS task. Recently, similar findings have been reported from a large-scale study of musical training and SOS masking (among several other measures; Whiteford, 2023). In that study, which used similar materials and methods as the current study, musicians had lower spatially separated thresholds by roughly 7 dB (more than 70 participants in each group, musicians and nonmusicians). The musicians as a group also scored significantly better than the nonmusician group on the RAPM, and the thresholds in the spatially separated condition (one spatial separation of ±15°) were predicted overall (across both groups) by performance on the RAPM.

Overall, the findings from the present study coupled with those of Clayton *et al.* (2016) and Whiteford (2023) suggest that cognitive/executive function abilities are related to success in cocktail party listening environments. The literature is not fully in accord with respect to which cognitive functions are most closely related to “speech in noise” performance, and the studies to date have not examined certain real-world factors, such as expectation and predictability, that almost certainly are key to individual differences. Nonetheless, the pattern emerging from these studies is that cognitive abilities are very important for successful communication in high-IM listening environments but are much less important in high EM environments. The relationship between cognitive abilities (as determined by problem-solving or recall tasks) and auditory IM is quite reasonable given that IM is attributed to limitations in central processing abilities rather than the fidelity of peripheral/sensory coding (Kidd and Colburn, 2017).

## VII. SUMMARY AND CONCLUSIONS

(1) For 18 normal hearing participants, individual differences were observed for spatial tuning of speech identification using various types of maskers, as well as for an analogous visual search task using distractor/masker objects with varying degrees of similarity to the target object. The general pattern of results was similar across modalities.
(2) Overall speech identification performance was positively correlated with a measure of participants' general cognitive reasoning, the number of years of musical training of the participants, and their overall performance on the visual search task. Visual task performance was also correlated with cognitive scores, but not musical training.
(3) Colocated SOS thresholds were correlated with SOS spatial filter bandwidth, suggesting that individuals with less susceptibility to colocated IM can also more easily separate speech streams with narrower spatial separations.
(4) The trends within the data support the conclusion that individuals with better performance for masked speech identification (in general) have sharper spatial tuning with less susceptibility to colocated SOS IM, in addition to higher cognitive reasoning skills.
(5) The ability of an individual to solve IM tasks may depend on mechanisms—such as various cognitive and executive functioning abilities—that are somewhat generalizable across different sensory modalities.
